# Single Administration of HBK-15—a Triple 5-HT_1A_, 5-HT_7_, and 5-HT_3_ Receptor Antagonist—Reverses Depressive-Like Behaviors in Mouse Model of Depression Induced by Corticosterone

**DOI:** 10.1007/s12035-017-0605-4

**Published:** 2017-05-26

**Authors:** Karolina Pytka, Monika Głuch-Lutwin, Magdalena Kotańska, Anna Waszkielewicz, Agnieszka Kij, Maria Walczak

**Affiliations:** 1 Department of Pharmacodynamics, Faculty of Pharmacy, Jagiellonian University Medical College, Medyczna 9, 30-688 Krakow, Poland; 2 Department of Pharmacobiology, Faculty of Pharmacy, Jagiellonian University Medical College, Medyczna 9, 30-688 Krakow, Poland; 3 Department of Bioorganic Chemistry, Chair of Organic Chemistry, Faculty of Pharmacy, Jagiellonian University Medical College, Medyczna 9, 30-688 Krakow, Poland; 4 Chair and Department of Toxicology, Faculty of Pharmacy, Jagiellonian University Medical College, Medyczna 9, 30-688 Krakow, Poland; 5 Jagiellonian Centre for Experimental Therapeutics, Bobrzyńskiego 14, 30-348 Krakow, Poland

**Keywords:** 5-HT_1A_ receptor antagonist, 5-HT_7_ receptor antagonist, 5-HT_3_ receptor antagonist, Corticosterone-induced model of depression, Pharmacokinetics, Blood–brain barrier, CD-1 mice

## Abstract

Studies suggest that the blockade of 5-HT_1A_, 5-HT_7_, and 5-HT_3_ receptor may increase the speed of antidepressant response. 1-[(2,6-Dimethylphenoxy)ethoxyethyl]-4-(2-methoxyphenyl)piperazine hydrochloride (HBK-14) and 1-[(2-chloro-6-methylphenoxy)ethoxyethyl]-4-(2-methoxyphenyl)piperazine hydrochloride (HBK-15), dual 5-HT_1A_ and 5-HT_7_ antagonists, showed significant antidepressant- and anxiolytic-like properties in our previous tests in rodents. In this study, we aimed to investigate their antidepressant potential using mouse model of corticosterone-induced depression. We chose sucrose preference test, forced swim test, and elevated plus maze to determine anhedonic-, antidepressant-, and anxiolytic-like activities. We also evaluated the influence of the active compound on brain-derived neurotrophic factor (BDNF) and nerve growth factor (NGF) levels in the hippocampus. Moreover, for both compounds, we performed biofunctional (5-HT_3_ receptor) and pharmacokinetic studies. We found that HBK-14 and HBK-15 were potent 5-HT_3_ receptor antagonists. HBK-14 (2.5 mg/kg) and HBK-15 (1.25 mg/kg) after intravenous (*i.v.*) and intraperitoneal (*i.p.*) administration permeated the blood–brain barrier with brain/plasma ratio lower than 1. The bioavailability of studied compounds after *i.p.* administration was 15% for HBK-14 and 54% for HBK-15. Chronic administration of HBK-15 (1.25 mg/kg) and fluoxetine (10 mg/kg) protected corticosterone-treated mice from anhedonic-, depressive-, and anxiety-like behaviors, as well as decreases in BDNF and NGF levels in the hippocampus. HBK-14 (2.5 mg/kg) counteracted anxiety-like behaviors in corticosterone-treated mice. Single administration of HBK-15 (1.25 mg/kg) and ketamine (1 mg/kg) reversed depression-like behavior and regulated decreased BDNF level in the hippocampus in corticosterone-treated mice. Our results suggest that simultaneous blockade of serotonergic 5-HT_1A_, 5-HT_7_, and 5-HT_3_ receptors might accelerate antidepressant response.

## Introduction

Depression is a prevalent, highly debilitating mental disorder, in which exact neurobiological mechanisms remain unknown. Antidepressants, even those recently discovered, are effective in only half of the patients. Moreover, the clinical response occurs following weeks to months of treatment. Thus, scientists still search for new, fast-acting antidepressants.

Although many systems participate in antidepressant-like effect (reviewed in [1–3]), most antidepressants interact with serotonergic system. Therefore, compounds that affect serotonin receptors are very interesting targets for researchers. Except for 5-HT_5_ receptor (which role in mood disorders is yet to be determined), most serotonin receptors take part in antidepressant-like response (for review, see Pytka et al. [1]). Given the localization and regulatory functions, 5-HT_1A_ receptors play significant role in mood regulation. The activation of presynaptic 5-HT_1A_ autoreceptors, expressed in the raphe nuclei, reduces the firing of serotonin neurons and consequently decreases serotonin release. On the other hand, postsynaptic heteroreceptors, highly expressed in limbic areas, such as hippocampus [4], regulate the release of other neurotransmitters (e.g., γ-aminobutyric acid or glutamate) [5, 6]. Scientists showed an increase in 5-HT_1A_ autoreceptors in postmortem brains from depressed suicide victims [7]. Similarly, animal studies proved that 5-HT_1A_ receptor deficient mice were less immobile in the forced swim test and tail suspension test compared with wild-type controls [8, 9]. Richardson-Jones and colleagues [10] demonstrated that mice, with lower 5-HT_1A_ autoreceptor levels before treatment, displayed a robust behavioral response to fluoxetine after both chronic and subchronic administration. The authors concluded that increasing serotonergic tone prior treatment with selective serotonin reuptake inhibitors might be more efficacious and even faster acting than current antidepressant therapies [10]. Similar conclusions, but concerning 5-HT_7_ receptors, drew Mnie-Filali and colleagues [11], who suggested that 5-HT_7_ receptor antagonists may represent a new class of antidepressant with faster therapeutic action. The scientists demonstrated that 1-week treatment with SB-269970 (a 5-HT_7_ receptor antagonist) caused behavioral, electrophysiological, and neuroanatomical changes that usually occur after long-term treatment with selective serotonin reuptake inhibitors [11].

Increasing the speed of antidepressant response might also be possible by blocking 5-HT_3_ receptors (for review, see Gupta et al. [12]). The 5-HT_3_ receptor differs structurally and functionally from all other serotonin receptors. This ligand-gated ion channel is cation-selective and mediates neuronal depolarization and excitation. Preclinical experiments suggested that 5-HT_3_ receptors blockade might contribute to vortioxetine’s faster onset of action [13]. Moreover, scientists proved that 5-HT_3_ receptor antagonism is a significant component of the drug’s antidepressant effect [13, 14]. Similar results obtained Eisensamer and colleagues [15], who proved that different classes of antidepressants act as functional antagonists at the human 5-HT_3A_. Moreover, studies on animals indicated that 5-HT_3_ receptor antagonists alleviate depressive- and anxiety-like behaviors in rodents [16–20].

Our previous experiments demonstrated that 1-[(2,6-dimethylphenoxy)ethoxyethyl]-4-(2-methoxyphenyl)piperazine hydrochloride (HBK-14) and 1-[(2-chloro-6-methylphenoxy)ethoxyethyl]-4-(2-methoxyphenyl)piperazine hydrochloride (HBK-15) were serotonin 5-HT_1A_ (IC_50_ = 64 nM—HBK-14, IC_50_ = 19 nM—HBK-15) and 5-HT_7_ (IC_50_ = 77 nM—HBK-14, IC_50_ = 220 nM—HBK-15) receptor antagonists with significant antidepressant- and anxiolytic-like activities in behavioral tests in rodents [21]. HBK-15 possessed moderate affinity for dopaminergic D_2_ (Ki = 54) and low for serotonergic 5-HT_2A_ (Ki = 109) receptors [21]. Although the compound moderately antagonized D_2_ (IC_50_ = 68 nM) and very weakly 5-HT_2A_ receptors (IC_50_ = 4459 nM), it did not show antipsychotic-like or cataleptogenic properties (unpublished results). HBK-14 and HBK-15 presented negligible cholinolytic [22] and antihistaminic activities [23]. We proved that despite α_1_-adrenolytic properties [24], neither HBK-14 nor HBK-15 lowered blood pressure at antidepressant- and anxiolytic-like doses after chronic treatment [23]. HBK-15 did not exhibit hypotensive activity even after single administration [24]. Interestingly, unlike most antidepressants, the compound showed memory-enhancing properties and ameliorated memory deficits induced by scopolamine in mice [23]. Both compounds administered chronically increased serotonin levels in murine hippocampus [22].

Considering the above data, we hypothesized that HBK-14 and HBK-15 as 5-HT_1A_ and 5-HT_7_ receptor antagonists, showing antidepressant- and anxiolytic-like activities in behavioral tests in rodents, might have potential to reverse behavioral abnormalities in depression model. Therefore, the aim of our study was to investigate antidepressant- and anxiolytic-like effects of the compounds using mouse model of corticosterone-induced depression. We also evaluated the effect of the active compound on brain-derived neurotrophic factor (BDNF) and nerve growth fact﻿or (NGF) levels in murine hippocampus, and determined if the compounds interact with serotonin 5-HT_3_ receptors. Since pharmacological activity of every compound depends on its absorption, distribution, metabolism, and excretion (ADME) properties, we also investigated the compounds’ pharmacokinetic properties.

## Materials and Methods

### Animals

Adult male mice (CD-1, 18–21 g) purchased from the Animal House at the Faculty of Pharmacy, Jagiellonian University Medical College, Krakow, Poland or male guinea pigs (Outbred CV, 300–400 g), purchased from Laboratory Animals Husbandry Maria Staniszewska, Słaboszów, Poland, were used in the experiments. Animals were kept to a plastic cage (mice 25.2 cm × 16.7 cm × 14.0 cm, four per cage; guinea pigs 60 cm × 380 cm × 20 cm, two per cage) at a room temperature (22 ± 2 °C) on 12 h light/dark cycles (the lights on at 7:00 a.m. and off at 19:00 p.m.). Animals had free access to standard laboratory pellet and tap water. All behavioral procedures were performed between 9 a.m. and 2 p.m. After the experiments, mice were killed by cervical dislocation, whereas guinea pigs were anesthetized (37 mg/kg sodium pentobarbital) and killed by cervical dislocation.

For pharmacokinetic study, HBK-14 and HBK-15 dissolved in saline were administered by an intravenous (*i.v.*) and intraperitoneal (*i.p.*) administration at a dose of 2.5 and 1.25 mg/kg, respectively. Blood samples were collected at 0 min (predose), 5 min, 15 min, 30 min, 60 min, 120 min, and 240 min after compounds administration. The blood and brain samples were collected under general anesthesia induced by *i.p*. injections of 50 mg/kg ketamine plus 8 mg/kg xylazine. Blood samples were collected into heparinized tubes. The samples were immediately centrifuged at 3500 rpm for 10 min, and plasma was collected. The brain and plasma samples were immediately frozen at −80 °C for further analysis.

All experimental procedures were carried out in accordance with EU Directive 2010/63/EU and approved by the I Local Ethics Committee for Experiments on Animals of the Jagiellonian University in Krakow, Poland (approval numbers: 52/2014, 123/2015, 261/2015, and 104/2016)*.*

### Drugs

**Fig. 1:**
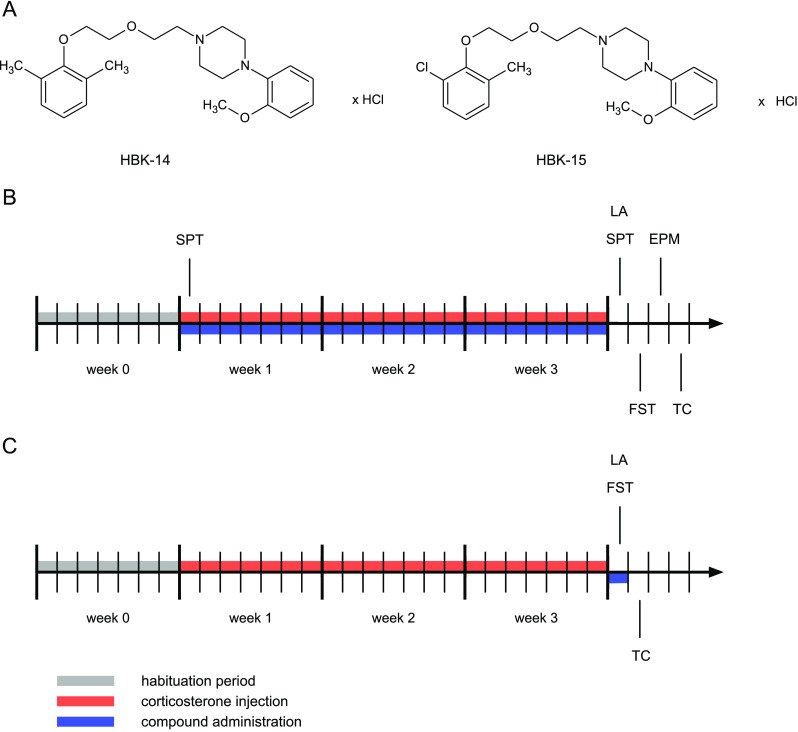
Chemical structures of the studied compounds and schematic diagrams of experimental procedures. **a** 1-[(2,6-Dimethylphenoxy)ethoxyethyl]-4-(2-methoxyphenyl)piperazine hydrochloride (*HBK-14*), 1-[(2-chloro-6-methylphenoxy)ethoxyethyl]-4-(2-methoxyphenyl)piperazine hydrochloride (*HBK-15*). **b** Corticosterone (20 mg/kg) was injected subcutaneously (*s.c.*) for 3 weeks at random times during the light phase. Additionally, 30 min before, corticosterone administration mice were intraperitoneally (*i.p.*) injected with HBK-14 (1.25 or 2.5 mg/kg), HBK-15 (0.625 or 1.25 mg/kg), fluoxetine (10 mg/kg), or 0.9% NaCl (saline). Control groups received saline (*i.p.*) and 30 min later saline containing 0.1% dimethyl sulfoxide (DMSO) and 0.1% Tween-80 (vehicle, *s.c.*). **c** Corticosterone (20 mg/kg) was injected *s.c.* to mice for 3 weeks at random times during the light phase. Thirty minutes before the experiment, mice were *i.p.* injected with HBK-15 (1.25 mg/kg), ketamine (1 mg/kg), fluoxetine (10 mg/kg), or 0.9% NaCl (saline). Control group, which was injected for 21 days with saline containing 0.1% dimethyl sulfoxide (DMSO) and 0.1% Tween-80 (vehicle, *s.c.*), received saline (*i.p.*). *EPM* - elevated plus maze, *FST* - forced swim test, *LA* - locomotor activity, *SPT* - sucrose preference test, *TC* - tissue collection

**Fig. 2:**
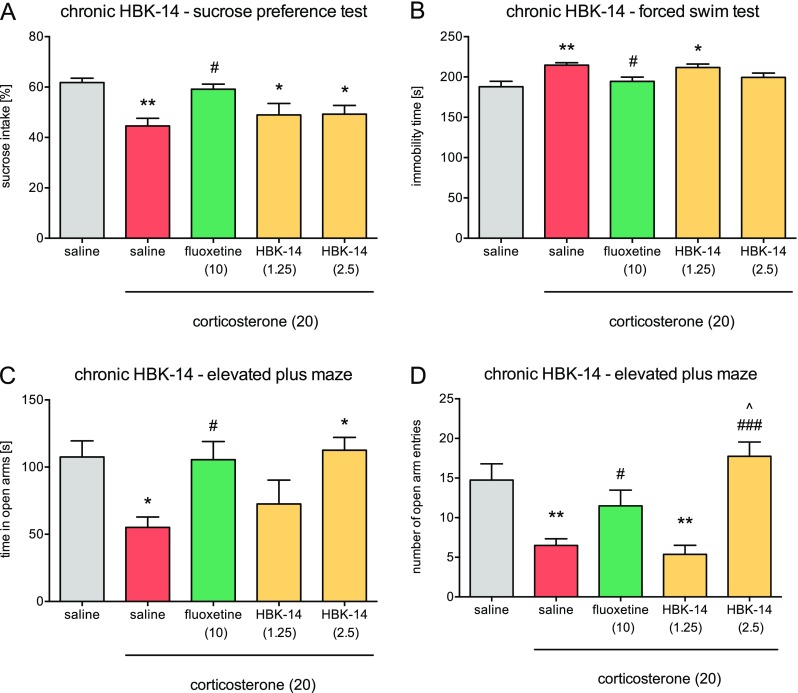
Effect of chronic administration HBK-14 and fluoxetine on the behavior of mice treated with corticosterone. Corticosterone (20 mg/kg) was injected subcutaneously (*s.c.*) for 3 weeks at random times during the light phase. Additionally, 30 min before, corticosterone administration mice were intraperitoneally (*i.p.*) injected with HBK-14 (1.25 or 2.5 mg/kg), fluoxetine (10 mg/kg), or 0.9% NaCl (saline). Control groups received saline (*i.p.*) and 30 min later saline containing 0.1% dimethyl sulfoxide (DMSO) and 0.1% Tween-80 (vehicle, *s.c.*). The doses are indicated in *brackets*. Statistical analysis: one-way ANOVA (Newman–Keuls post hoc); *p < 0.05, **p < 0.01 vs control (non-corticosterone-treated) (saline, *gray*); ^#^ *p* < 0.05, ^###^ *p* < 0.001 vs corticosterone-treated control (saline, *red*); ^^^ *p* < 0.05 vs fluoxetine; *n* = 8 mice per group (color figure online)

1-[(2,6-Dimethylphenoxy)ethoxyethyl]-4-(2-methoxyphenyl)piperazine hydrochloride (HBK-14; Fig. 1a) and 1-[(2-chloro-6-methylphenoxy)ethoxyethyl]-4-(2-methoxyphenyl)piperazine hydrochloride (HBK-15; Fig. 1a) were synthesized in the Department of Bioorganic Chemistry, Chair of Organic Chemistry, Pharmaceutical Faculty, Jagiellonian University [25]. HBK-14, HBK-15, fluoxetine (Sigma, Germany), and ketamine (Sigma, Germany) were dissolved in saline, and administered *i.p.* at a volume of 10 mL/kg. Corticosterone was dissolved in saline containing 0.1% dimethyl sulfoxide (DMSO) and 0.1% Tween-80 and administered subcutaneously (*s.c.*). Serotonin (Sigma, Germany) was dissolved in distilled water and used in biofunctional studies.

### The Effect on Guinea Pig Ileum Contractions Induced by Serotonin

The experiment was performed according to the method described by Mogilski and colleagues [26]. A segment (15 cm) of male guinea pig ileum was excised from the small intestine and immersed into a Krebs solution (NaCl 120 mM, KCl 5.6 mM, MgCl_2_ 2.2 mM, CaCl_2_ 2.4 mM, NaHCO_3_ 19 mM, glucose 10 mM). The part of the ileum (5 cm) that was the closest to the ileo-cecal junction was removed. After 2-cm-long fragments of the ileum were cut, each of them was placed in 20 mL chamber of tissue organ bath system (Tissue Organ Bath System—750 TOBS, DMT, Denmark) filled with the Krebs solution at 37 °C, pH 7.4, with constant oxygenation (O_2_/CO_2_, 19:1). The segments were stretched by means of closing clips between the metal rod and the force–displacement transducer. The preparations were stabilizing in organ baths for 60 min under a resting tension of 0.5 g and were washed every 15 min with fresh Krebs solution. After the equilibration period, a cumulative concentration–response curve was constructed for serotonin (3 nM–3 μM). Then, the tissues were incubated with one of the concentrations of tested compounds for 15 min, and the next cumulative concentration curve to the agonist was constructed. Only one concentration of the potential antagonist was tested in each piece of the tissue. The experiment was repeated four to eight times.

### Experimental Design

#### Chronic Administration of Studied Compounds

The doses and route of administration of the studied compounds were based on our previous experiments [21, 22]. Since antidepressant-like activity of HBK-14 and HBK-15 was due to the interaction with serotonergic (not adrenergic) system [21], we chose fluoxetine (selective serotonin reuptake inhibitor) as reference drug. Mice were injected with corticosterone (*s.c.*, 20 mg/kg) at random times during the light phase for 21 consecutive days (Fig. 1b). The dose and route of administration of corticosterone was based on studies performed by Zhao and colleagues [27]. Thirty minutes before, corticosterone injection mice were given (*i.p.*) saline, HBK-14 (1.25 or 2.5 mg/kg), HBK-15 (0.625 or 1.25 mg/kg), or fluoxetine (10 mg/kg) (Fig. 1b). Control mice received (*i.p.*) saline and 30 min later saline containing 0.1% dimethyl sulfoxide (DMSO) and 0.1% Tween-80 (*s.c.*). The experimental groups consisted of eight randomly selected animals. After the end of the procedure, animals were tested in the sucrose preference test, forced swim test, elevated plus maze test, and spontaneous locomotor activity test. Following behavioral testing, rodents were scarified and their hippocampi collected for biochemical analysis.

#### Acute Administration of Studied Compounds

Mice were injected with corticosterone (*s.c.*, 20 mg/kg) at random times during the light phase for consecutive 21 days (Fig. 1c). The dose and route of administration of corticosterone were based on studies performed by Zhao and colleagues [27]. After the end of the procedure, next day 30 min before the test, corticosterone-treated mice were given (*i.p.*) saline, HBK-15 (1.25 mg/kg), fluoxetine (10 mg/kg), or ketamine (1 mg/kg) (Fig. 1c). Control mice received (*i.p.*) saline. The experimental groups consisted of eight randomly selected animals. We chose the dose of HBK-15 that was active in the chronic experiments (i.e., 1.25 mg/kg). The dose of ketamine was based on the studies performed by Pazini and colleagues [28]. Animals were tested in the forced swim test and spontaneous locomotor activity test. After behavioral testing, mice were scarified and their hippocampi collected for biochemical analysis.

### Spontaneous Locomotor Activity

The locomotor activity was performed as previously described [29]. Locomotor activity was recorded individually for each mouse using activity cages made of clear Perspex (40 cm × 40 cm × 31 cm, Activity Cage 7441, Ugo Basile, Italy). The cages were supplied with I.R. horizontal beam emitters connected to a counter for the recording of light-beam interruptions. Each mouse was placed in a cage for a 30-min habituation period. After that time, the number of crossings of photobeams was measured for 6 min (chronic studies) or for 4 min (acute experiments; i.e., the time equal to the observation period in the forced swim test). The cages were disinfected with 70% ethanol after each mouse.

### Sucrose Preference Test

The sucrose preference test was conducted according to the slightly modified methods described by Liu and colleagues [30] and Filho and colleagues [31]. The test was performed before and after the administration of corticosterone. Briefly, before the proper test, mice were housed singly and given 72 h training to acclimate to the test procedures. In the first 24 h, access ad libitum to two feeding bottles of 1% (*w*/*v*) sucrose solution was given to each mouse. After 24 h, one bottle was replaced with tap water for the next 24 h. Then, mice were deprived of water and food for the third 24 h. On the test day, the weight of each bottle (1% sucrose solution and tap water) was recorded. Test animals were then given the bottles of 1% sucrose and water for 24 h. The consumed liquid weight was measured based on the weight of each bottle of fluids after sucrose preference test minus the original starting weight. The percentage of sucrose intake was calculated using the following equation:

$$ \%\mathrm{Sucrose}\ \mathrm{intake}=\frac{\mathrm{Sucrose}\ \mathrm{intake}}{\mathrm{Sucrose}\ \mathrm{intake}+\mathrm{water}\ \mathrm{intake}}\cdot 100\% $$

### Forced Swim Test

Forced swim test was performed according to the method described by Porsolt and colleagues [32] and previously described [33, 34]. Mice were placed individually for 6 min in glass cylinders (height 25 cm, diameter 10 cm) containing 10 cm^3^ of water (23–25 °C). The total time of immobility was recorded during the final 4 min of the test. The experiments were recorded and scored using aLab.io software by a trained observer blind to the treatments.

### Elevated Plus Maze

The elevated plus maze was performed according to the method previously described [35, 36]. The elevated plus maze for mice consisted of two opposing open (30 cm × 5 cm), and two enclosed arms (30 cm × 5 cm × 25 cm) connected by a central platform forming the shape of a plus sign. The open and closed arms were connected with a central field (5 cm × 5 cm). Each mouse was individually placed at the central field of the apparatus with the head turned toward one of the closed arms. Animal behavior was observed for 5 min. The device was disinfected with 70% ethanol after each mouse. The number of entries to open and closed arms and time spent in the open and closed arms were measured. The experiments were recorded and scored using aLab.io software by a trained observer blind to the treatments.

### BDNF and NGF Levels in the Hippocampus

After the behavioral assessments, mice were sacrificed, and their brains were rapidly removed and chilled in an ice-cold saline solution. The hippocampi were dissected on a cold plate, frozen, and stored at −80 °C until assay. On the day of experiments, tissues were thawed on ice and homogenized (1:9 *w*/*v*) in phosphate-buffered saline (4 °C) and protease inhibitor cocktail was added. The 10% homogenates were prepared and homogenized for 30 s with TissueRuptor homogenizer. The homogenized tissues were centrifuged (2500×*g* at 4 °C for 20 min), and the supernatants were collected for further assays.

Protein concentrations of BDNF and NGF in homogenates from hippocampi were determined using the enzyme-linked immunosorbent assay (ELISA) kits (BDNF: DZE201020014, SunRed Biotechnology Company; NGF: MBS825100, MyBioSource) according to the manufacturer’s instructions. Serial dilutions of the standards were performed to make the standard curve within the range of this assay (BDNF 0.1–10 ng/mL; NGF 31.2–2000 pg/mL). The samples were analyzed in duplicates, and the mean concentrations were calculated. BDNF and NGF antibodies are high selectivity and thus did not cross-react with any other cytokines. The reaction was terminated after the stop solution was added. The intensity of the color was read at 450 nm. Absorbance was measured in a multifunction plate reader (POLARstar Omega, BMG Labtech, Germany). The concentration of the samples was interpolated from the standard curve using GraphPad Prism Version 6.00 software.

### Pharmacokinetic Studies

Pharmacokinetic parameters were calculated by a non-compartmental approach from the average concentration values, using Phoenix WinNonlin software (Certara, Princeton, NJ 08540, USA). First-order elimination rate constant (*λ*_*z*_) was calculated by linear regression of time vs log concentration. Next, the area under the mean serum and brain concentration vs time curve extrapolated to infinity (AUC_0→∞_) was estimated using the log-linear trapezoidal rule (Eq. 1), where *C*_*n*_ is the concentration of last sampling of each compound.

1 $$ {\mathrm{AUC}}_{0\to \infty }=\sum_{i=1}^n\left(\left({C}_i+{C}_{i+1}\right)/2\right)\cdot \left({t}_{i+1}-{t}_i\right)+{C}_n/{\lambda}_z $$

Area under the first moment curve (AUMC_0→∞_) was estimated by calculation of the total area under the first moment curve and extrapolated area using the Eq. 2, where *t*_*n*_ is the time of last sampling.

2 $$ {\mathrm{AUMC}}_{0\to \infty }=\sum_{i=1}^n\left(\left({t}_i\cdot {C}_i+{t}_{i+1}\cdot {C}_{i+1}\right)/2\right)\cdot \left({t}_{i+1}-{t}_i\right)+\left({t}_n\cdot {C}_n\right)/{\lambda}_z+{C}_n/{\lambda}_z^2 $$

Mean residence time (MRT) was calculated as

3 $$ \mathrm{MRT}=\frac{{\mathrm{AUMC}}_{0\to \infty }}{{\mathrm{AUC}}_{0\to \infty }} $$

Total clearance (Cl_T_) was calculated as

4 $$ {\mathrm{Cl}}_{\mathrm{T}}=\frac{D_{\mathrm{iv}}}{{\mathrm{AUC}}_{0\to \infty }} $$

Volume of distribution at steady state (V_ss_) was calculated as

5 $$ {\mathrm{V}}_{\mathrm{ss}}=\frac{D_{\mathrm{iv}}\cdot {\mathrm{AUMC}}_{0\to \infty }}{{{\mathrm{AUC}}_{0\to \infty}}^2} $$

The bioavailability (*F*) of HBK-14 and HBK-15 after *i.p.* administration was calculated as follows

6 $$ F=\frac{{\mathrm{AUC}}_{\mathrm{i}.\mathrm{p}.}}{{\mathrm{AUC}}_{\mathrm{i}.\mathrm{v}.}}\cdot 100\% $$

where *D*_iv_ is an *i.v.* dose of HBK-14 and HBK-15, AUC is the area under the zero moment curve, and AUMC is the area under the first moment curve.

#### Analytical Procedure

The quantification of studied compounds in plasma and brain samples was accomplished using UFLC Nexera system (Shimadzu, Kyoto, Japan) coupled to the triple quadrupole mass spectrometer QTrap 5500 (Sciex, Framingham, MA, USA) equipped with Turbo V™ ion source. After preparation, the samples were injected (5 μL) onto Acquity UPLC BEH C18 (3.0 × 100 mm, 1.7 μm, Waters, Milford, MA, USA) analytical column. The mobile phases that consisted of ACN with 0.1% FA (A) and water with 0.1% FA (B) were delivered in isocratic elution mode (40% A, 60% B) at the flow rate of 550 μL/min. The total time of analysis was 8 min including 2 min of column equilibration.

Electrospray ionization process was performed in positive polarization, and the data acquisition was carried out in multiple reaction monitoring mode (MRM) for all analytes and their internal standard (HBK-11 [37]). The ion spray source settings were as follows: spray voltage 4.5 kV, heater temperature 450 °C, curtain gas 25 psi, source gas 1 40 psi, source gas 2 40 psi. All manually optimized parameters for each analyte including ion transitions and collision energy (CE) were listed in Table 1.

**Table 1:** The ion transitions selected for studied compounds quantification and internal standard registration as well as all manually optimized parameters including collision energy (CE), declustering potential (DP), entrance potential (EP), and collision cell exit potential (CXP)

Analyte	MRM (*m*/*z*)	CE [V]	DP [V]	EP [V]	CXP [V]
HBK-14	385.2 → 205.3	38	140	15	10
HBK-15	405.2 → 190.3	38	100	15	20
IS (HBK-11)	431.2 → 239.2	39	80	15	25

#### Sample Preparation

An amount of 50 μL of sample (plasma or brain homogenate) was transferred into the clean Eppendorf tube and spiked with 5 μL of internal standard solution (IS, 1 μg/mL) obtaining the final concentration of 100 ng/mL. After 5 min of mixing (1500 rpm), proteins were precipitated using 150 μL ACN. After 10 min of samples shaking (1500 rpm), the incubation step was performed (10 min, 4 °C). Next, samples were centrifuged (10,000 rpm, 10 min, 4 °C) and the supernatant was transferred into chromatographic vial for LC/MS/MS analysis.

The brain homogenate was prepared maintaining the tissue: phosphate-buffered saline (PBS) ratio at 1:5. The homogenization was carried out employing IKA® T10 Basic ULTRA-TURRAX disperser (IKA Werke GmbH & Co. KG, Staufen, Germany). After homogenization, all samples were centrifuged (3000 rpm, 10 min, 4 °C) and 50 μL of supernatant was collected for further preparation. All samples were stored on ice during the preparation process.

#### Standard Solution Preparation

Each studied compound in amount of 5 mg was accurately weighted and quantitatively transferred into the 5-mL volumetric flask using MeOH. After the salts dissolving flask was filled to the 5 mL mark with MeOH obtaining 1 mg/mL of particular analyte. Further dilutions were performed using MeOH to prepare working standard solutions of analytes at the following concentrations: 0.025, 0.05, 0.1, 0.25, 0.5, 1.0, 2.5, 5.0, 10, 25, and 50 μg/mL for calibration curve (CC) samples and 0.025, 0.075, 2.2, and 4.5 μg/mL for quality control (QC) samples.

A volume of 45 μL of matrix (plasma or brain homogenate) was spiked with 5 μL of IS obtaining the final concentration of 100 ng/mL and 5 μL of standards working solutions at needed CC or QC concentration levels. After standard solution addition, samples were mixed and purified in the same way as unknown (studied) samples.

### Data Analysis

Results are presented as means ± S.E.M. The comparisons between experimental and control groups were performed by one-way ANOVA, followed by Newman–Keuls post hoc (GraphPad Prism version 6.00 software). A value of *p* < 0.05 was considered to be significant.

In functional experiments p*K*_B_ values for non-competitive antagonists were estimated using double-reciprocal plot method and the following equation [38]

$$ \begin{array}{l}\mathrm{p}{K}_{\mathrm{B}}=-{ \log}_{10}\frac{\left[ B\right]}{\mathrm{slope}-1}\\ {}\left[ B\right]\hbox{--} \mathrm{molar}\ \mathrm{antagonist}\ \mathrm{concentration}\end{array} $$

## Results

### HBK-15 and HBK-14 Decreased Guinea Pig Ileum Contractions Induced by Serotonin

Serotonin concentration dependently contracted guinea pig ileum; the pEC_50_ value (negative logarithm of the agonist concentration at which the response reached 50% of the maximal response) was 5.95 ± 0.07. None of the studied compounds administered alone had effect on ileal contractions (data not shown). HBK-14 at the concentrations 10, 30, and 100 nM decreased the maximum effect of serotonin by 24, 49, and 65%, respectively. This suggested a non-competitive antagonism. HBK-15 at the concentrations 30, 100, and 300 nM decreased the maximal response by 24, 55, and 65%, respectively, which indicated a non-competitive interaction with 5-HT_3_ receptors. The p*K*_B_ values are presented in Table 2.

**Table 2:** Functional affinities of HBK-14 and HBK-15 for 5-HT_3_ receptors expressed in guinea pig ileum

Compound	5-HT_3_ receptor p*K*_B_ ± S.E.M
HBK-14	7.949 ± 0.10
HBK-15	7.361 ± 0.12
Ondansetron	7.111 ± 0.12^a^

^a^[26]

### Chronic Experiments

#### Chronic Treatment with HBK-15 but Not HBK-14 Prevented Anhedonic-Like Behavior in Corticosterone-treated Mice

**Fig. 3:**
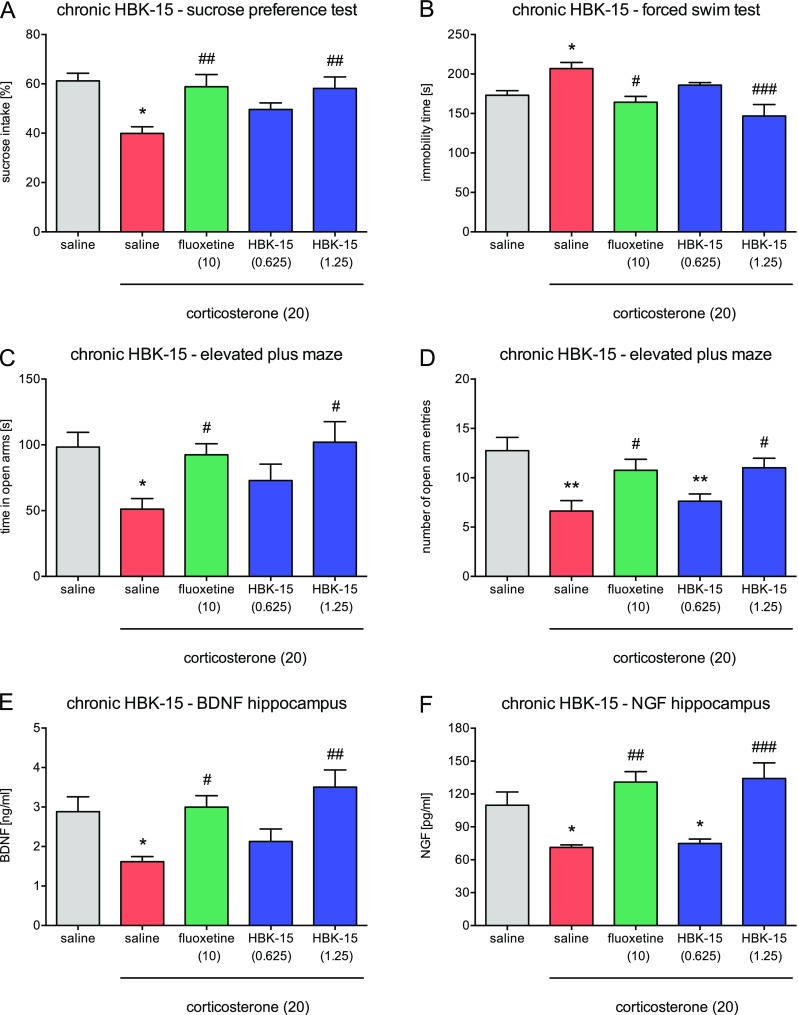
Effect of chronic administration HBK-15 and fluoxetine on corticosterone-treated mice behavior (**a**–**c**) and BDNF (**d**) and NGF (**e**) levels in the hippocampus. Corticosterone (20 mg/kg) was injected subcutaneously (*s.c.*) for 3 weeks at random times during the light phase. Additionally, 30 min before, corticosterone administration mice were intraperitoneally (*i.p.*) injected with HBK-15 (0.625 or 1.25 mg/kg), fluoxetine (10 mg/kg), or 0.9% NaCl (saline). Control groups received saline (*i.p.*) and 30 min later saline containing 0.1% dimethyl sulfoxide (DMSO) and 0.1% Tween-80 (vehicle, *s.c.*). The doses are indicated in *brackets*. Statistical analysis: one-way ANOVA (Newman–Keuls post hoc); *p < 0.05, **p < 0.01 vs control (non-corticosterone-treated ) (saline, *gray*); ^#^ *p* < 0.05, ^##^ *p* < 0.01, ^###^ *p* < 0.001 vs corticosterone-treated control (saline, *red*); *n* = 8 mice per group (animal studies), *n* = 6 mice per group (biochemical studies) (color figure online) *BDNF* - brain-derived neurotrophic factor, *NGF* - nerve growth factor

There were no significant differences in sucrose intake between the studied groups at the beginning of the experiment [HBK-14 *F*(4,35) = 0.288, ns; HBK-15 *F*(4,35) = 0.132, ns] (data not shown). After 21 days in mice injected with corticosterone (20 mg/kg) receiving saline, the percentage intake of sucrose solution was significantly decreased (by 17%—HBK-14, Fig. 2a or 21%—HBK-15, Fig. 3a) compared with control animals. HBK-14 was inactive in this test [*F*(4,35) = 4.678; *p* < 0.01] (Fig. 2a). HBK-15 (1.25 mg/kg but not 0.625 mg/kg) and fluoxetine (10 mg/kg) administered for 21 days prevented the decrease in sucrose intake in mice treated with corticosterone [*F*(4,35) = 5.483; *p* < 0.01] (Fig. 2a).

#### Chronic Treatment with HBK-15 but Not HBK-14 Prevented Depressive-Like Behavior in Corticosterone-treated Mice

We observed a significant increase (by 14%—HBK-14, Fig. 2b or 21%—HBK-15, Fig. 3b) in the immobility in mice treated with corticosterone (20 mg/kg) receiving saline compared with controls. HBK-14 was inactive in this test [*F*(4,35) = 5.030; *p* < 0.01] (Fig. 2b). HBK-15 (1.25 mg/kg but not 0.625 mg/kg) and fluoxetine (10 mg/kg) administered for 21 days prevented an increase in immobility in mice treated with corticosterone [*F*(4,35) = 6.984; *p* < 0.01] (Fig. 3b).

#### Chronic Treatment with HBK-15 and HBK-14 Prevented Anxiety-Like Behavior in Corticosterone-treated Mice

Mice treated with corticosterone (20 mg/kg) receiving saline compared with controls spent significantly less time (HBK-14: by 48%, Fig. 2c; HBK-15: by 48%, Fig. 3c) in the open arms and entered the open arms less often (HBK-14: by 56%, Fig. 2d; HBK-15: by 48%, Fig. 3d). HBK-14 (2.5 mg/kg but not 1.25 mg/kg) and fluoxetine (10 mg/kg) prevented the decreases in the time spent in the open arms [*F*(4,35) = 4.029; *p* < 0.01], and the number of open arm entries [*F*(4,35) = 10.490; *p* < 0.0001] in mice treated with corticosterone (Fig. 2c, d). HBK-14 (2.5 mg/kg) significantly increased the number of open arms entries compared with fluoxetine (Fig. 2d). HBK-15 (2.5 mg/kg but not 1.25 mg/kg) and fluoxetine (10 mg/kg) prevented the decreases in the time spent in the open arms [*F*(4,35) = 3.394; *p* < 0.05], and the number open arm entries [*F*(4,35) = 5.675; *p* < 0.01] in corticosterone-treated mice (Fig. 3c, d).

#### None of the Studied Compounds Administered Chronically Influenced Locomotor Activity of Corticosterone-Treated Mice

There were no differences between corticosterone-treated mice receiving saline and controls (Table 3). Neither HBK-14 (1.25 and 2.5 mg/kg), HBK-15 (0.625 and 1.25 mg/kg), nor fluoxetine (10 mg/kg) administered for 21 days influenced locomotor activity in 6 min session [HBK-14 *F*(4,35) = 0.384, ns; HBK-15 *F*(4,35) = 0.371, ns] in corticosterone-treated mice (Table 3).

**Table 3:** Effect of chronic treatment with studied compounds on locomotor activity in mice

Treatment	Dose of the studied compound (mg/kg)	Number of crossings ± S.E.M
Saline + vehicle	–	366.0 ± 59.3
Saline + corticosterone	–	343.4 ± 27.3
HBK-14 + corticosterone	1.25	406.3 ± 64.2
HBK-14 + corticosterone	2.5	400.8 ± 55.8
Fluoxetine + corticosterone	10	423.3 ± 47.3
Saline + vehicle	–	436.9 ± 71.7
Saline + corticosterone	–	394.5 ± 67.1
HBK-15 + corticosterone	0.625	466.1 ± 84.8
HBK-15 + corticosterone	1.25	478.9 ± 78.8
Fluoxetine + corticosterone	10	510.3 ± 54.0

Corticosterone (20 mg/kg) was injected subcutaneously (*s.c.*) for 3 weeks at random times during the light phase. Additionally, 30 min before, corticosterone administration mice were intraperitoneally (*i.p.*) injected with HBK-14 (1.25 or 2.5 mg/kg), HBK-15 (0.625 or 1.25 mg/kg), fluoxetine (10 mg/kg), or 0.9% NaCl (saline). Control groups received saline (*i.p.*) and 30 min later saline containing 0.1% dimethyl sulfoxide (DMSO) and 0.1% Tween-80 (vehicle, *s.c.*). Statistical analysis: one-way ANOVA (Newman–Keuls post hoc); *n* = 8 mice per group

#### Chronic Treatment with HBK-15 Prevented Corticosterone-Induced Decrease in BDNF Level in Murine Hippocampus

There was a significant decrease in BDNF level (by 44.0%) in the hippocampus in mice treated with corticosterone (20 mg/kg) receiving saline compared with controls (Fig. 3e). HBK-15 (1.25 but not 0.625 mg/kg) and fluoxetine (10 mg/kg) administered for 21 days protected corticosterone-treated mice from the decrease in the BDNF level in the hippocampus [*F*(4,25) = 5.286; *p* < 0.01] (Fig. 3e).

#### Chronic Treatment with HBK-15 Prevented Corticosterone-Induced Decrease in NGF Level in Murine Hippocampus

The level of NGF in murine hippocampus was significantly lower (35%) in mice treated with corticosterone (20 mg/kg) receiving saline compared with controls (Fig. 3f). HBK-15 (1.25 but not 0.625 mg/kg) and fluoxetine (10 mg/kg) administered for 21 days protected corticosterone-treated mice from the decrease in the NGF level in the hippocampus [*F*(4,25) = 9.690; *p* < 0.0001] (Fig. 3f).

### Acute Experiments

#### Single Administration of HBK-15 Reversed Depressive-Like Behavior in Corticosterone-Treated Mice

**Fig. 4:**
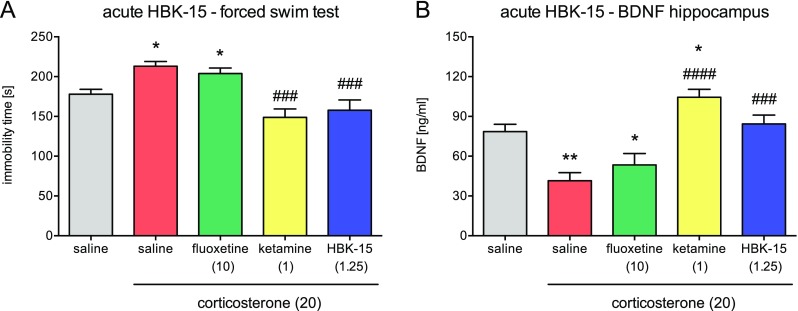
Effect of a single administration of HBK-15, fluoxetine, and ketamine on the immobility in the forced swim test (**a**) and BDNF level in the hippocampus (**b**) in corticosterone-treated mice. Corticosterone (20 mg/kg) was injected subcutaneously (*s.c.*) to mice for 3 weeks at random times during the light phase. Thirty minutes before the experiment, mice were intraperitoneally (*i.p.*) injected with HBK-15 (1.25), ketamine (1 mg/kg), fluoxetine (10 mg/kg), or 0.9% NaCl (saline). Control group, which was injected for 21 days with saline containing 0.1% dimethyl sulfoxide (DMSO) and 0.1% Tween-80 (vehicle, *s.c.*), received saline (*i.p.*). Statistical analysis: one-way ANOVA (Newman–Keuls post hoc); *p < 0.05, **p < 0.01 vs control (non-corticosterone-treated) (saline, *gray*); ^###^ *p* < 0.001, ^####^ *p* < 0.0001 vs corticosterone-treated control (saline, *red*); *n* = 8 mice per group (animal studies), *n* = 7 mice per group (biochemical studies) *BDNF* - brain-derived neurotrophic factor

We observed a significant increase (by 20%; Fig. 3b) in immobility in mice treated with corticosterone (20 mg/kg) receiving saline compared with controls. HBK-15 (1.25 mg/kg) and ketamine (1 mg/kg) but not fluoxetine (10 mg/kg) administered 30 min before the test reversed an increase in immobility in corticosterone-treated mice [*F*(4,35) = 9.783; *p* < 0.0001] (Fig. 4a).

#### Acute Treatment with HBK-15 Did Not Influence Locomotor Activity of Corticosterone-Treated Mice

We did not observe any differences between mice treated with corticosterone (20 mg/kg) for 21 days, which received saline and controls (Table 4). Single administration with HBK-15 (1.25 mg/kg), ketamine (1 mg/kg), or fluoxetine (10 mg/kg) did not influence locomotor activity [*F*(4,35) = 0.191, ns] of corticosterone-treated mice (Table 4).

**Table 4:** Effect of a single administration of studied compounds on locomotor activity in mice

Treatment	Dose of the studied compound (mg/kg)	Number of crossings ± S.E.M
Saline + vehicle	–	246.5 ± 34.6
Saline + corticosterone	–	230.7 ± 15.8
HBK-15 + corticosterone	1.25	245.3 ± 22.5
Ketamine + corticosterone	1	248.7 ± 25.3
Fluoxetine + corticosterone	15	231.9 ± 21.9

Corticosterone (20 mg/kg) was injected subcutaneously (*s.c.*) to mice for 3 weeks at random times during the light phase. Thirty minutes before, the experiment mice were intraperitoneally (*i.p.*) injected with HBK-15 (1.25), ketamine (1 mg/kg), fluoxetine (15 mg/kg), or 0.9% NaCl (saline). Control group, which was injected for 21 days with saline containing 0.1% dimethyl sulfoxide (DMSO) and 0.1% Tween-80 (vehicle, *s.c.*), received saline (*i.p.*). Statistical analysis: one-way ANOVA (Newman–Keuls post hoc); *n* = 8 mice per group

#### Single Administration of HBK-15 Reversed Corticosterone-Induced Decrease in BDNF Level in Murine Hippocampus

There was a significant decrease in BDNF level (by 47.0%) in the hippocampus in mice treated with corticosterone (20 mg/kg) for 21 days, which received saline compared with controls (Fig. 4b). Single administration of HBK-15 (1.25 mg/kg) but not fluoxetine (10 mg/kg) reversed the decrease in BDNF level in the hippocampus [*F*(4,30) = 3.690; *p* < 0.0001] (Fig. 4b). Acute treatment with ketamine increased (by 33%) the level of BDNF in the hippocampus in corticosterone-treated mice compared with controls (Fig. 4b).

### Pharmacokinetic Studies

**Fig. 5:**
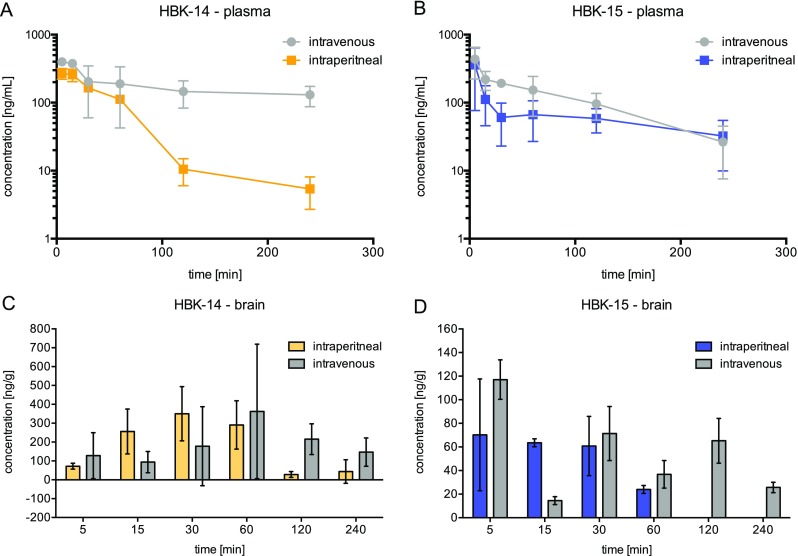
Concentration–time profile in plasma (**a**, **b**, semilogarithmic plots) and distribution to brain (**c**, **d**) for HBK-14 (2.5 mg/kg) and HBK-15 (1.25 mg/kg) after intravenous or intraperitoneal administration to mice

The mean plasma concentrations vs time for HBK-14 and HBK-15 after *i.v.* and *i.p.* administration are shown in Fig. 5a, b. The pharmacokinetic parameters calculated for both compounds by non-compartmental approach are given in Table 5. The compounds were cleared from mouse body with varying rate. After the *i.v.* and *i.p.* administration of HBK-14, the terminal half-lives were 323 and 38 min, respectively. For HBK-15, the terminal half-lives were 71 min after *i.v.* and 84 min after *i.p.* administration. The volume of distribution for HBK-14 was greater (11.2 L/kg) than adequate for HBK-15 (3.5 L/kg) that indicates the ability of these compounds to penetrate to the deep compartments. After *i.p.* administration, both compounds very quickly, within 5 min, reach the maximum concentration in the blood. HBK-15 reaches higher bioavailability (*F* = 54%) compared with HBK-14 (*F* = 15%). The investigated compounds penetrated the blood–brain barrier in a similar degree (Fig. 5c, d). After *i.v.* administration of HBK-14 and HBK-15, the brain/plasma ratio was 0.80 and 0.68, respectively, whereas after *i.p.* administration of HBK-14 and HBK-15, the brain/plasma ratio was 0.46 and 0.27, respectively. Distribution of HBK-15 to the brain was very rapid and the maximal concentration occurred 5 min after *i.p.* administration (Fig. 5d).

**Table 5:** Pharmacokinetic parameters for HBK-14 (2.5 mg/kg) and HBK-15 (1.25 mg/kg) after *i.v.* and *i.p.* administrations

Parameters	HBK-14		HBK-15	
	*i.v.*	*i.p.*	*i.v.*	*i.p.*
*C*_0_ [ng/mL]	410	–	602	–
AUC_0→∞_ [ng min/mL]	103,499	33,617	29,018	15,683
MRT [min]	454.1	48.3	90.2	108.7
*t*_0.5_ [min]	322.7	38.2	70.5	83.5
*C*_max_ [ng/mL]	–	269.3	–	359
*t*_max_ [min]	–	5	–	5
*V*_d_ [L/kg]	11.2	–	3.5	–
Cl [mL/min/kg]	24.2	–	40.2	–
*F* [%]	15		54	

Twenty-eight adult male mice (CD-1, 20–25 g) were used in the study

*C*_*0*_ the initial concentration, *C*_*max*_ the maximum plasma concentration, *t*_*max*_ time to reach the maximum plasma concentration, *t*_*0.5*_ terminal half-life, MRT mean residence time, *AUC*_*0→∞*_ area under the concentration–time curve from zero up to infinitive time, *Cl* systemic clearance, *V*_*d*_ volume of distribution at steady state, *F* absolute bioavailability, *i.v.* intravenous, *i.p.* intraperitoneal

## Discussion

We found that a single administration of HBK-15—a triple 5-HT_1A_, 5-HT_7_, and 5-HT_3_ receptor antagonist—reversed depression-like behavior and regulated decreased BDNF level in the hippocampus in mice with corticosterone-induced depression model. Chronic treatment with the studied compound protected corticosterone-treated mice from anhedonic-, depressive-, and anxiety-like behaviors, as well as decreases in BDNF and NGF levels in the hippocampus. A structural analogue (HBK-14); however, administered chronically showed only anxiolytic-like activity. HBK-15 showed desirable ADME profiles.

Although the data is inconsistent, most reports suggest the potential role of 5-HT_3_ antagonists in the treatment of depression (reviewed in [12]). Moreover, some studies show that 5-HT_3_ receptor blockade might result in anxiolytic-like effect [39, 40]. Bearing that in mind, we tested HBK-14 and HBK-15 in biofunctional assays to evaluate their effect on 5-HT_3_ receptors. Our results indicate that both compounds were non-competitive 5-HT_3_ receptor antagonists. We think that 5-HT_3_ receptor antagonism might have contributed to the antidepressant- and anxiolytic-like effects of the compounds.

Pharmacokinetic properties of compounds influence their pharmacological action. Therefore, we investigated the pharmacokinetic profiles of HBK-14 (2.5 mg/kg—active dose) and HBK-15 (1.25 mg/kg— active dose) after *i.v.* and *i.p.* administration in mice. The concentration of target compounds in plasma was determined using LC/ESI-MS/MS system. If 50 μL of plasma was used, concentrations of both compounds were detectable for at least 4 h after administration. The pharmacokinetic results showed that the absorption of both compounds was rapid, with the peak concentration occurring at 5 min after *i.p.* administration. HBK-15 showed higher bioavailability (*F* = 54%) compared with HBK-14 (F = 15%). HBK-14 compared with HBK-15 demonstrated higher volume of distribution, 11.2 vs 3.5 L/kg. Both compounds penetrated the blood–brain barrier but the ratio of the concentrations in the brain-to-plasma was less than 1. Considering the pharmacokinetic study and brain penetration, we conclude that HBK-15 presents desirable ADME profile to follow preclinical studies.

Corticosterone-induced model of depression is a widely used preclinical model. The repeated administration of corticosterone induces behavioral (e.g., reduced sucrose consumption [41] or increased immobility in the forced swim test [42]) and neurochemical (e.g., decreased neurogenesis in the hippocampus [43, 44]) changes in rodents that mimic some core symptoms of depression [45]. Antidepressants reverse these changes [28]. Here, we demonstrated that mice chronically injected with corticosterone showed anhedonic- (reduced sucrose consumption), depressive- (increased immobility in the forced swim test), and anxiety-like (decreased number of entries and time spent in the open arms of the elevated plus maze) behaviors. HBK-14 (2.5 mg/kg but not 1.25 mg/kg) administered for 21 days prevented anxiolytic-like behavior, whereas HBK-15 (1.25 mg/kg but not 0.625 mg/kg) and fluoxetine protected corticosterone-treated mice from all above behaviors. We think that the lower dose of HBK-15 was inactive in this model, because it was insufficient to induce neurochemical changes necessary for antidepressant- and anxiolytic-like effects. The effect of the higher dose was comparable to fluoxetine. Since none of the treatments affected locomotor activity of mice, the observed results were specific.

Interestingly, HBK-14 prevented corticosterone-treated mice only from anxiety-like behavior. Although anxiolytic-like effect (the increase in the number of open arm entries) was significantly stronger than that of fluoxetine, HBK-14, unlike HBK-15, failed to protect mice from depressive-like behaviors. We suggest two possible explanations of this phenomenon. First, it might be due to the slight differences in the receptor profiles of the compounds—HBK-14, compared with HBK-15, is a weaker 5-HT_1A_ and stronger 5-HT_7_ receptor antagonist (the effect on 5-HT_3_ receptors is comparable) [21]. According to Wesołowska and colleagues [46], the potent 5-HT_7_ receptor blockade reduced anxiety-like symptoms in rodents. Since HBK-14 is stronger 5-HT_7_ receptor antagonist, its anxiolytic-like effect might be more profound. These findings are consistent with our previous experiments, where we demonstrated that among the two, HBK-14 possessed stronger anxiolytic-like properties, whereas HBK-15 antidepressant-like activity [21, 22]. The second explanation is simply the difference in bioavailability—HBK-14 has lower bioavailability, and this might underlie the lack of antidepressant-like effect. Perhaps low bioavailability is the reason why we observed only the stronger, anxiolytic-like, component of the compound’s activity. Nevertheless, to fully understand this issue, we need to perform further studies.

Our previous experiments revealed that chronic treatment with HBK-15 increased serotonin level in the hippocampus [22]. Serotonin, at least partially, regulates the level of neurotrophins in the central nervous system [47, 48]. Neurotrophins, such as BDNF or NGF, play important role in the adaptation of neural networks (including neurogenesis) that are responsible for different aspects of mood regulation and antidepressant-like effect (reviewed in [49]). Numerous studies reported reduced BDNF and NGF levels in patients with mood disorders [50–53]. Preclinical studies confirm that low levels of BDNF and NGF result in depressive-like behaviors in rodents. Advani and colleagues [54] demonstrated that a deficiency in BDNF made male mice vulnerable to mild stress and increased signs of behavioral despair in the forced swim test (immobility), as well as plasma corticosterone levels. Other authors proved that a reduction in BDNF expression in the dentate gyrus reduced neurogenesis and affected behaviors associated with depression [55]. Moreover, a specific BDNF knockdown in the ventral subiculum induced anhedonic-like behavior [55]. Similarly, several animal studies showed lower NGF levels in the hippocampus and prefrontal cortex in chronically stressed rodents [31, 56–58], as well as antidepressant-like effect of NGF itself [59, 60].

Taking the above data into account, we decided to investigate the influence of HBK-15 on BDNF and NGF levels in corticosterone-treated mice. Consistent with other studies [28], we showed that corticosterone administration decreased both neurotrophins levels in murine hippocampus. Similar to fluoxetine, HBK-15 (1.25 but not 0.625 mg/kg) administered chronically protected corticosterone-treated mice form these decreases. Since most antidepressants upregulate neurotrophins levels after chronic treatment, this feature of HBK-15 is beneficial.

Considering the receptor profile and pharmacological activity of HBK-15, we decided to test its antidepressant potential after single administration in corticosterone-induced model of depression. Our results indicate that, similar to ketamine, HBK-15 (1.25 mg/kg) reversed depressive-like behavior, without influencing locomotor activity of animals. Fluoxetine was inactive after a single administration. As mentioned in the “Introduction” section, the blockade of presynaptic 5-HT_1A_ autoreceptors, as well as 5-HT_7_ and 5-HT_3_ receptors, might contribute to or even accelerate antidepressant effect. We speculate that HBK-15 selectively blocked presynaptic 5-HT_1A_ autoreceptors, which combined with 5-HT_7_ and 5-HT_3_ receptor blockade, increased the speed of antidepressant-like response. Nevertheless, our theory needs confirmation, as this effect might be a result of the interaction with other systems. We also demonstrated that a single administration of HBK-15 (like ketamine but to a lesser extent) upregulated BDNF level in the hippocampus. Most antidepressants increase BDNF levels and stimulate neurogenesis, but this effect occurs after several weeks of administration (maturation of new neurons). Moreover, we previously reported memory-enhancing properties of HBK-15 [22]. Since none of the current antidepressants show rapid clinical effect and significantly improve cognitive function, our results are very promising.

The limitation of our study was that we did not examine the precise mechanism underlying the rapid action of HBK-15 in corticosterone-induced model of depression. Therefore, in future studies, we plan to investigate the mechanisms by which HBK-15 exerted its fast effects. Moreover, since depression is more prevalent in women than in men, we should also evaluate the activity of HBK-15 in female mice.

## Conclusion

We demonstrated that a single administration of HBK-15—a triple 5-HT_1A_, 5-HT_7_, and 5-HT_3_ receptor antagonist—reversed depression-like behavior and regulated decreased BDNF level in the hippocampus in mouse with corticosterone-induced model of depression. Chronic treatment with the studied compound protected corticosterone-treated mice from anhedonic-, depressive-, and anxiety-like behaviors, as well as deceased BDNF and NGF levels in the hippocampus. We think that simultaneous 5-HT_1A_, 5-HT_7_, and 5-HT_3_ receptor blockade might accelerate antidepressant response, and therefore, HBK-15 requires extended studies to explore its full pharmacological profile.
